# Cases of Cerebro-Spinal Meningitis in Post Hospital, Gallop’s Island, Boston Harbor, from September, 1864, to May, 1865

**Published:** 1865-10

**Authors:** Calvin G. Page


### CASES OF CEREBRO-SPINAL MENINGITIS IN POST HOSPITAL, GALLOP'S ISLAND, BOSTON HARBOR, FROM SEPTEMBER, 1864, TO MAY, 1865.

By CALVIN G. PAGE, M.D.

[Reported to the Boston Society for Medical Improvement, June 12th, 1865, and communicated for the Boston Medical and Surgical Journal.]

Case I. L. B. L., recruit, from Pittsfield, Mass., 18 years old, light complexion, blue eyes, and of bilious, sanguine temperament. Was admitted to hospital Sept. 14th, having been unwell for two days previous to reporting at sick call. When first seen, patient was complaining of severe headache, attended with extreme weakness; had been vomiting bilious matter, and had had several evacuations of thick mucus of the consistence of gruel; no pain in the bowels; tongue was badly coated and flabby; pulse at wrist barely perceptible; patient stupid, but not delirious. I ordered hot baths, during which purple spots of irregular shapes appeared on the abdomen, chest, and thighs, increasing in size and color very rapidly; extremities cold and shriveled. By hot applications externally, and stimulants internally, reaction commenced in three hours from the bath, and with it delirium, exhibiting itself by continued incoherent talking as long as the strength lasted, ending in a whisper at the last breath.

Sept. 15th.—Noticed rigidity of the muscles of the neck, with extreme tenderness of spine in lumbar region, and tendency to roll from one side to the other; very restless, constantly talking, and momentarily conscious when spoken to. I noticed, this day, an eruption about the mouth and nose of an erysipelatous nature, the purple spots rather tending to recede or dry, with a circumscribed base, as if about to slough, which did not occur, but they gradually disappeared by the fifth day from their appearance. There was no marked change in patient from this time to his death, which occurred on the 19th. Opisthotonos increased to the last, yielding to no treatment. Allowing the disease to have commenced on the 12th, two days previous to his entry to the hospital, the length of the attack was seven days.

Case II. E. D., recruit, from South Reading, Mass., 15 years old, dark complexion, slight in person, and not a healthy looking boy. Patient was admitted January 29th, complaining of excessive pain in the head, of cold running down the back, and sickness at stomach, vomiting thick bilious matter. Cold applications were made to the head, and blisters to the spine and neck.

Jan. 30th.—Complains of pain "all over;" very sore to the touch, and no diminution of pain in head; is inclined to coma. Face, to-day, is flushed, and pulse 120, slightly tremulous. Blisters drew well; also had a free evacuation from the bowels, but without result. Symptoms rather tending to congestion, which proved to be the case on the following day, as the patient died comatose, and with stertor in breathing towards the close. There was no decided opisthotonos in this case, nor petechiæ, but from the extreme sensitiveness of touch and extent of pain in the head, I could class it under no other head.

CASE III. B. F. W., recruit, from Boston, 15 years old, slight in person, and not of robust constitution. Was admitted to hospital Jan. 9th, 1865, complaining of sickness at stomach, severe pain in the head, and aching in every joint. The whole surface had a peculiar mottled appearance, being much like a faint eruption of measles, the spots more generally diffused, however, than in rubeola; having measles in the hospital at the time, I concluded to put him in the measles ward. Patient was delirious at times during the day, and very restless; pulse 130.

Jan. 10th.—Could perceive no eruption; pulse still 130, and irregular. Had him put in the main ward. No abatement of pain; still delirious and very restless.

11th.—Patient seems brighter, but no better; the pulse increasing and tremulous; face livid; head very hot, with decided symptoms of congestion; no opisthotonos; pupils dilated; breathing labored. Patient died in the early part of the afternoon. Like the preceding case, I could class it under no other head, although the symptoms were indistinct.

CASE IV. O. W., recruit, from near New Bedford, 19 years old, a stout, robust, healthy person. The only defect found at time of examination, was a dry, hard, callous-like ulcer on the bottom of the heel, which, from his own account, discharged occasionally a watery fluid, causing no pain, nor in any way interfering with his daily avocation as a teamster. January 28th, patient came to sick call, feeling slight headache and tired. I ordered cinchonated whiskey, and excused him from duty.

Jan. 29th.—Reported this morning as feeling much the same; had vomited during the night, from overloading the stomach with sweetcake and pie. In the afternoon of the same day, the sergeant came to the hospital, stating that the patient was much worse, and was turning purple. I ordered him brought to the hospital immediately. On his arrival, I found the whole surface covered with petechiæ; patient insensible, and evidently too far gone to rescusitate. No morbid symptoms in this case until within thirty minutes of his death.

CASE V. L. C., an old soldier, from Plymouth, Ct., 30 years old, was sent to this post for transportation, convalescent from some disease, I know not what. I was called to see the patient in barracks, as having a fit. Found no paroxysm, but a constant tumbling from right to left side, he unable to speak and seemingly unconscious. I ordered him to hospital, and administered chloroform with the usual effect weile under its influence. Patient vomited bile, with no change of symptoms, and was so restless as to require strapping to the bed. Symptoms yielded slightly under large doses of opium, producing, however, no real relief. He died the day following of congestion. No opisthotonos or petechiæ, but emprosthotonos, with decided cerebral trouble of some kind.

Case VI. A. A. M., recruit, from Cambridgeport, Mass., 19 years old, stout and healthy, of sandy complexion, a teamster. Came to sick call, feeling unfit for duty, but not really sick. Gave no medicine. In the afternoon, came to hospital feeling sick at stomach, with slight drowsiness of head. Gave an emetic, which had, seemingly, a favorable effect. This was at at 4 P.M. At 6, I examined patient and found him, to use his own words, feeling every way comfortable, but cold. I could discover no pulse at the wrist, and extremities were stone cold. I changed his bed to one beside the stove, to which he walked with perfect ease. Could discover no petechiæ, although the steward noticed them when he first came in. No opisthotonos, but decided symptoms of congestion. He died at 9 that evening, having the stertorous breathing similar to the preceding cases.

Case VII. J. E. S., an old soldier, from Concord, N. H., 22 years old, florid complexion, of nervous, sanguineous temperament; subject to epilepsy. Patient was brought here from the boat, having been taken while *en route* for this post. Symptoms were, excessive pain in the head and limbs, and prostration; no vomiting or petechiæ. I applied leeches to the temples, and gave oleum ricini, ℥ss., ol. croton, tiglii, gtt. iij. This was February 15th. The day following, patient complained less of pain in the head, but felt stiffness in cords of neck. Made use of stimulating liniment to neck, and gave pil. hydrarg. gr. vij, at bedtime.

17th.—Is feeling better, but very weak. Ordered stimulating diet, and continued the liniment. On the 20th, patient was able to sit up; still very weak.

21st.—Patient complained more of stiffness of neck and joints of lower extremities; is feverish and wandering, inclining to whistle rather than to talk. Could perceive no swelling of joints, and but slight contraction of muscles; tongue coated and not easily managed. Patient remained much the same for some days. On the 26th, noticed a difficulty in swallowing and a hesitation in answering questions; head more inclined to left side, evincing great pain when moved; countenance troubled, and had a peculiar stare when spoken to; evacuations from bowels and bladder involuntary, although regular and natural. From this date, patient gradually sank, with no change in character of disease, and died March 17th, 1865, four weeks and two days from first attack. Could discover no petechiæ in this case, but decided opisthotonos of the entire left side, with complete prostration, was present from Feb. 21st. Patient had gonorrhœa when admitted, which continued to the time of his death; also slight swelling of testicle in the early part of the attack.

CASE VIII. O. B., old soldier, 20 years old, stout, and to all appearance healthy. Was admitted to hospital February 8th, 1865. Complained of excessive pain in head, neck, and side, to which blisters were applied, and pil. hydrarg., gr. vij, was given at night.

9th.—Head and neck still very painful, patient shrinking from the slightest motion of either. Ordered liniment to neck; also quinia, grs. v., Dover's powder, grs. vij., every four hours.

10th.—The same. Continued treatment. Tongue thickly coated, but moist.

11th.—Ordered pil. hydrarg., grs. vij., at night. Liniment. Patient complains less, but still shrinks from pain; is at times delirious. I used beef-tea and egg punch freely.

16th.—Patient allows himself to be moved, although very sore and weak. As in the last case, the head inclines to the left shoulder, and symptoms every way resemble that case. Patient is of much stronger constitution, and was free from disease of any kind. Patient was discharged from hospital, Mar. 2d, convalescent.

CASE IX. J. P., old soldier, from Providence, R. I., 17 years old, rather small and immature, but apparently healthy. Was admitted to hospital, February 10th, 1865, at the time comparatively helpless, with involuntary discharges from bowels and vomiting of bilious matter. Petechiæ were seen on the abdomen, with muscular contractions from head to heels. Delirious most of the time. Blisters were applied to neck and spine, hot stones to feet, and quinia sulph., grs. vij., capsicum, grs. ij., whiskey ℥ij., every three hours during the day. Dover's powder, grs. vij., morph. sulph., gr. ¼, at bedtime.

11th.—Muscles of neck very tense, and head so drawn back as to obstruct the œsophagus. This continued for three days, during which time injections of beef-tea and wine, each one pint, were given every three hours, nights excepted. On the fifth day from the attack, the muscles of the neck relaxed a trifle, and patient could, with great effort, swallow a little; in doing which, copious discharges of dark, tenacious mucus were produced. From this date, patient continued to improve on stimulating diet alone.

March 1st.—Noticed swelling of parotid gland, attended with pain and loss of appetite, tongue coated, and feverish symptoms accompanying. I gave potass. chl. sol., freely painting the neck with tinct. iodine at night. A full month was embraced in subduing this attack, patient being able to walk by means of crutches.

May 1st.—Acute rheumatism set in, attended with swelling of joints and difficulty of breathing, probably the effects of sudden cold. No fever, no disturbance of bowels, no coating of the tongue, and yet patient could not be moved or touched without screaming. I gave sol. potass. iodidi during the day, and one-fourth of a grain of morphia at bedtime. Generous diet. At present time patient is able to sit up and stand, but not to walk. His chances seem good for recovery. I should have stated that deafness attended the first attack.

Case X. J. B. F., recruit, from Berkshire County, Mass., 18 years old, not a healthy person. Admitted February 18th, 1865, complaining of chills, weakness and slight headache. Gave oleum ricini, ℥ss. Afternoon.—Still complains of feeling cold. Gave quinia, gr. vij., capiscum, gr. ij., whiskey, ℥ij. and ordered a hot bath, which brought out spots on the face and body. Pulse barely perceptible; extremities very cold, and countenance anxious; mind perfectly clear. In all cases thus far the spots have been angular, of no particular shape, and generally from the size of a small bean to that of a chesnut. The prominent symptom in this case was the sensation of cold, as expressed by the patient's constantly stepping out of bed to go to the stove, which he did thirty minutes before he died, death taking place twelve hours from the attack. No opisthotonos, or pain, except in head, and no amount of heat made any impression upon the chilliness. The dilatation of the pupils was very great.

Case XI. C. I., recruit, from New Ipswich, N.H., 18 years old, robust, healthy, and every way sound. Was admitted February 23d, complaining of headache and complete blindness. Tongue coated, and stinging pains in arms and legs. Gave oleum ricini, ℥ss., ol. croton. tiglii. gtt. iij. Noticed spots on arms, legs, and face; also noticed redness of the eyes. In one hour's time the spots, which were more circular than angular, increased from the size of beans to large patches, running together, especially on the forearms, growing very dark and sore to the touch. Pulse feeble; extremities cold, but patient expressed himself as feeling comfortable, if let alone.

24th.—Spots still very dark and swollen, showing a disposition to slough, having every appearance of sores from caustic potash.

25.—Line of demarkation very distinct, especially on the arms and wrist. On the 26th, skin sloughed out, leaving the whole inner surface of forearm down to fascia raw and excessively painful, suppurating freely. Spots on legs formed thick crusts, discharging matter from the under surface continually. From the commencement of the sloughing, patient improved slowly until near the first of April, when sore mouth and tongue set in, attended by profuse expectoration, similar to ptyalism. This subsided in a fortnight's time, and patient improved rapidly in every respect, excepting the eyesight, which only allowed him to discern objects independently of form.

May 11th.—Patient received his discharge from the U. S. service, which prevented any further observation of the case. There was no opisthotonos in this case, or cerebral disturbance. Secretions and evacuations every way natural and healthy.

Case XII. A. S. P., recruit, from Gardner, Me., 21 years old, very robust and healthy. Was brought to hospital early in the morning of March 20th, insensible, helpless, and very restless; head inclined backwards, but muscles not rigid; tongue badly coated and brown; face very red, and pulse high.

22d.—Eruption about the face, more particularly the mouth and nose; could discover no spots, nor did the patient seem sensible to pain. Patient would pass his water involuntarily, and most of the time was delerious. Symptoms continued bad from the commencement to the time of his death, April 8, 1865.

Case XIII. Dr. G. A. P., recruit, from Jaffrey, N.H., 40 years old, a practising physician, came to this post from Concord, N. H., having attended a case of spotted fever previous to coming here. Excessive pain in the head and limbs; prostration; purple spots on the abdomen, making their appearance on the third day from the first sensation of illness. Patient was admitted to hospital April 11th. Neck stiff, very sore to the touch and inclined to left side. No change in symptoms until April 13th, when the face, directly in front of right ear commenced to swell, and by night had increased up and down, from the temple to the clavicle, embracing the parotid gland, very hard and extensive, and not seemingly painful.

15th.—Pus commenced discharging from the ear, very freely. No fluctuation in the tumor.

17th.—An incision was made at the base of the tumor, also another on the day following, followed by free discharge. During the first two weeks patient seemed perfectly indifferent, would answer indirectly, with momentary consciousness. Patient continued to improve from the commencement of the discharge from the abscess. Most of the time had no control of rectum or bladder.

May 7th.—Was conveyed home convalescent.

CASE XIV. J. R., recruit, from Boston, Mass., 38 years old, of delicate constitution, sailmaker by trade. Was admitted March 22d, with pains in whole body; felt so sore as not to bear his weight; cramped all over; inclined to be stupid. No spots perceptible, nor were there any other prominent symptoms. His breathing was stertorous previous to his death, which occurred March 30th, eight days from attack.

CASE XV. A. W. F., recruit, from Bangor, Me., musician, 19 years old, stout and healthy. Symptoms as in previous cases. Was admitted April 3d. Pulse very high; neck stiff, and although sensible, could not speak; countenance troubled, and patient very restless. Sodæ bisulph. was given freely, and continued, with no perceptable effect.

4th.—Pulse 100; face flushed, and paralysis of left side.

5th.—Stertorous breathing, with livid countenance. Patient died in the afternoon. No spots or opisthotonos.

CASE XVI. W. L., recruit, from Hopkinton, Mass., farmer, 19 years old, was at the time of attack in hospital for measles. Admitted March 6th.

10th, 4 P. M.—Spots came out all over the body; no headache; mind clear. In two hours the whole surface was nearly black, in which state patient died, complaining only of difficulty in breathing. Patient occupied the bed previously used by Case VIII. Whether the disease had any other than a peculiar turn and terminus of measles I cannot say; it certainly differed from anything I ever witnessed. The spots were isolated, and wholly independent of the rubeola.

CASE XVII. W. M., recruit, from Roxbury, Mass., 19 years old, healthy. Was admitted March 7th. Died March 10th, having symptoms like the previous cases. Petechiæ on abdomen. No opisthotonos.

Other cases of a mild character have occurred, but hardly deserving mention. I would remark that in all cases, mild or exaggerated, erysipelatous eruptions have occurred on the third or fourth day from attack; and in all cases where the patient was conscious, soreness of the limbs and feet has prevailed, and in most cases involuntary discharges from the rectum and bladder. In but two instances could anything like contagion be traced.

ANALYSIS OF CASES.

| | *Amitted.* | *Died.* | |
|---|---|---|---|
| 1. L. B. L. | Sept. 14th, 1864. | Sept. 19th. | |
| 2. C. B. | Jan. 25th, 1865. | Jan. 27th. | |
| 3. E. D. | " 29th, " | " 31st. | |
| 4. O. W. | " " " | " 29th. | |
| 5. L. C. | Feb. 6th, " | Feb. 7th. | |
| 6. O. B. | " 8th, " | | Recovered. |
| 7. B. F. W. | " 9th, " | " 11th. | |
| 8. J. P. | " 10th, " | | Recovered. |
| 9. A. A. M. | " " " | " 10th. | |
| 10. E. F. | " 14th, " | " 15th. | |
| 11. J. E. S. | " 15th, " | March 17th. | |
| 12. J. B. F. | " 18th, " | Feb. 18th. | |
| 13. W. L. | March 6th, " | March 10th. | |
| 13. W. M. | " 7th, " | " " | |
| 15. A. S. P. | " 20th, " | April 8th. | |
| 16. J. R. | " 22d, " | March 30th. | |
| 17. J. F. P. | " 29th, " | April 16th. | |
| 18. A. W. F. | April 3d, " | " 5th. | |
| 19. G. A. P. | " 11th, " | | Recovered. |

